# Comparison of Different Ultrasound Protocols in Patients with Inflammatory Polyneuropathies with Practical Insights

**DOI:** 10.3390/diagnostics15121484

**Published:** 2025-06-11

**Authors:** Evelina Grusauskiene, Agne Smigelskyte, Erisela Qerama, Daiva Rastenyte

**Affiliations:** 1 Department of Neurology, Medical Academy, Lithuanian University of Health Sciences, LT-44307 Kaunas, Lithuania; 2 Department of Clinical Neurophysiology, Aarhus University Hospital, 8200 Aarhus, Denmark

**Keywords:** peripheral nerve ultrasound, ultrasound protocols, UPSS, Bochum, EAN/PNS CIDP recommendations

## Abstract

**Objectives**: We aimed to compare well-known ultrasound protocols for inflammatory polyneuropathies in a single cohort. **Methods**: High-resolution ultrasound was performed according to the Bochum ultrasound score (BUS)/neuropathy ultrasound protocol (NUP), ultrasound pattern sum score (UPSS), and EAN/PNS suggested protocol for patients with chronic (CIDP) and acute inflammatory polyneuropathies (AIDP), multifocal motor neuropathies (MMN) and healthy controls. The upper boundaries were adjusted according to our laboratory normative values to all above-mentioned protocols; additionally, another calculation was performed using the peripheral nerve size values officially proposed by EAN/PNS. **Results**: We enrolled a total of 189 subjects (105 males and 84 females), comprising 40 patients with CIDP, 13 with MMN, 11 with AIDP, and 125 healthy controls. The mean ages were 62.49 years (range 37–84 years) for the CIDP patients; 55.92 years (range 32–71 years) for the MMN patients; 68.09 years (range 51–88 years) for the AIDP patients; and 49.02 years (range 25–80 years) for the healthy controls. Using the EAN/PNS protocol bilaterally, 72.9% of CIDP cases were identified. When the adjusted EAN/PNS protocol was applied, the detection rate rose to 100%, with a sensitivity of 100%. Both the adjusted BUS/NUP and UPSS protocols demonstrated a specificity of 90% in diagnosing CIDP. EAN/PNS protocol detected 69.23% of MMN cases measured unilaterally and had a 100% sensitivity to distinguish MMN, while the UPSS protocol had the highest specificity (96%). In AIDP cases, the adjusted EAN/PNS protocol identified 90.90% of cases through unilateral or bilateral measurements, with sensitivity 91% and specificity 88%. **Conclusions**: The EAN/PNS protocol was the most valuable in the detection of treatable states, and the BUS/NUP, UPSS protocols were the most valuable in the differentiation of specific inflammatory polyneuropathies.

## 1. Introduction

Diagnostics of inflammatory polyneuropathies remain challenging despite all the modern methods used in daily practice. Although nerve conduction studies have been considered the gold standard, clinicians frequently encounter difficulties in establishing a definitive diagnosis. A number of studies have been performed on the application of peripheral nerve ultrasound in inflammatory disorders [1,2,3,4,5,6,7,8,9,10], including chronic inflammatory demyelinating polyneuropathy (CIDP) [11], multifocal motor neuropathy (MMN) [12], acute inflammatory demyelinating polyneuropathy (AIDP), and others [11]. High-resolution ultrasound (HRUS) is easy to perform, well tolerated by the patient, and has a low cost. A prospective multicenter study provided class IV evidence that their suggested nerve ultrasound protocol is an accurate diagnostic tool for detecting chronic inflammatory neuropathies with the sensitivity of 84.6% and specificity of 72.8% [10]. Recently, peripheral nerve ultrasound was included in EAN/PNS guidelines for the diagnosis and treatment of CIDP [13] as a supportive criterion in cases with diagnostic uncertainty. However, HRUS was not included in the EAN/PNS guidelines for the diagnosis and treatment of Guillain–Barré syndrome [14] due to the lack of established cut-off values for abnormal findings and the wide variability observed in diagnostic sensitivity and specificity across previous studies [15,16,17]. As a result, further research was recommended to better define the specificity of HRUS in AIDP patients [14]. Although some studies demonstrated the benefits of ultrasound for the diagnostic of the most of inflammatory polyneuropathies, only a few scanning protocols are suggested [13,17,18,19]. The ultrasound pattern sum score (UPSS), Bochum ultrasound score (BUS) [17], and extended version of BUS—neuropathy ultrasound protocol (NUP) [18] are probably the best-known and discussed protocols for inflammatory polyneuropathy diagnostics. BUS/NUP protocols might help identify CIDP, MMN, AIDP, and even one of the CIDP variants—multifocal acquired demyelinating sensory and motor neuropathy (MADSAM), also vasculitic neuropathy or paraproteinemia neuropathy [18]. The UPSS protocol was originally developed to distinguish AIDP from CIDP. Over time, it has also proven useful in identifying characteristic changes in other acquired neuropathies (MMN, MADSAM), as well as various hereditary and paraproteinemia polyneuropathies [20].

EAN/PNS recommendations for using HRUS in clinical practice for CIDP were developed based on studies that evaluated the diagnostic value of ultrasound in identifying various treatable inflammatory polyneuropathies [6,16,21,22].

The protocols vary in their extent of application: the BUS/NUP protocol is performed bilaterally in all cases, whereas the UPSS protocol is applied unilaterally on the right side in symmetric neuropathies. While EAN/PNS recommendations do not explicitly state whether bilateral measurements are required [7,10]. The aim of our study was to compare the sensitivity and specificity of established ultrasound protocols for inflammatory polyneuropathies in a single cohort, following the adjustment to our laboratory-specific reference values.

## 2. Methods

A case control study was conducted at the Department of Neurology of the Hospital of Lithuanian University of Health Sciences Kauno Klinikos (Kaunas, Lithuania) from April 2022 to October 2024.

The study was approved by the Kaunas Regional Biomedical Research Ethics Committee with bioethical permission No. BE-2–29, issued on 14 April 2022, which also covers the data used in related publications, in accordance with the ethical principles of the Declaration of Helsinki.

### 2.1. Selection of Participants

During April, 2022, to October, 2024, 82 patients diagnosed with inflammatory polyneuropathy at the Department of Neurology were invited to participate in the study. Thirteen patients were excluded because they did not meet the diagnostic criteria for the CIDP, MMN, or AIDP, and five patients with AIDP were excluded from the study due to their critical condition and severely limited mobility, which made a comprehensive ultrasound examination unfeasible.

Thus, 64 patients with diagnosed inflammatory polyneuropathy were recruited into the study. Additionally, 125 healthy subjects with an age range of 25–80 years were recruited for this study. The healthy control group consisted of medical staff and patients who were treated in the Department of Neurology for conditions that do not affect the peripheral nervous system (epilepsy, transient ischemic attack, stroke, and headache). Data collection for the healthy controls and the establishment of our laboratory reference values have been previously published [23].

All participants signed an informed consent form, and participation was entirely voluntary, without any honorarium received. Data on age, gender, results of nerve conduction studies (patients only), laboratory tests, and information regarding the disease course were collected for all subjects. All subjects underwent neurological examination and HRUS was performed as part of the study protocol.

The diagnosis of AIDP was made based on diagnostic criteria by the National Institute of Neurological Disorders and Stroke (NINDS) [24]. The diagnosis of MMN was made based on revised diagnostic criteria by the European Federation of Neurological Societies (EFNS) and Peripheral Nerve Society (PNS) (2010) [25]. The diagnosis of CIDP was based on diagnostic criteria by the European Academy of Neurology and Peripheral Nerve Society (EAN/PNS) (2021) [13].

### 2.2. Ultrasound Examination

The ultrasound examination of peripheral nerves was performed by a neurologist with two years of experience (EG) in neuro-muscular ultrasound. Philips EPIQ 7 ultrasound machine (Philips EPIQ Diagnostic Ultrasound System, Bothell, WA, USA, 2019) with a linear 4–18 MHz transducer (eL18-4, piezo elements 1920) in B mode was used for the nerve‘s cross-sectional area (CSA) assessment. Measurements were performed bilaterally. The precise methodology was described earlier [23], during establishing our laboratory‘s normative values.

We performed ultrasound scanning according to Bochum ultrasound score (BUS)/neuropathy ultrasound protocol (NUP) [18], ultrasound pattern sum score (UPSS) [11], and the protocol suggested by the EAN/PNS [13].

Briefly, according to BUS (step 1) the ulnar (UN) nerve was measured at Guyon’s canal and the upper arm, the radial nerve (RN) at the spiral groove, and the sural nerve (SN) at the calf. The BUS was extended by NUP (step 2), and median (MN) and UN nerve measurements were recorded in the middle of the forearm, the tibial nerve (TN) was measured at the ankle beneath the vascular arcade. MN at wrist and UN at elbow measurements were performed according to NUP (step 3).

UPSS protocol was performed according to its three sub-scores (UPS-A, UPS-B, and UPS-C). Following the UPS-A subscore, MN and UN measurements were performed in the midpoint of the upper arm, the midpoint of the forearm, and MN in the middle arm. The tibial nerve (TN) and fibular nerve (FN) were measured at the popliteal fossa. Additionally, the TN was measured at the ankle. According to UPS-B subscore, the diameters of the fifth (C5) and the sixth (C6) cervical roots were measured after leaving the intervertebral foramen, directly after the transverse process. The vagal nerve (VN) was measured at the level of the carotid triangle. Lastly, the UPS-C subscore’s measurements were performed, the sural nerve (SN) was measured in the calf, the superficial radial nerve was recorded at the level of the arcade of Frohse, and the fibular superficial fibular nerve was measured at the ankle.

As specified in the EAN/PNS guidelines for the diagnosis and treatment of CIDP [13] MN measurements were taken at the midpoint of the upper arm, and the midpoint of the forearm. Additionally, the upper (UT) and middle trunks (MT) of the brachial plexus were assessed in inter-scalene space and the CSAs of cervical roots C5 and C6 were measured. The upper boundary limits for the specified measurements according to BUS/NUP and UPSS were adjusted according to our laboratory’s normative values, taking into account sex differences. These adjusted protocols are referred to as BUS/NUP adj., UPSS adj. and EAN/PNS adj. in the results and discussion sessions, following the approach used in the original studies, and the term “EAN/PNS protocol” indicates that we used estimates suggested by official EAN/PNS guidelines (Table 1).

According to BUS/NUP protocol, one point was assigned if the CSA value exceeded the reference limits, and zero points were given if the value fell within the normal reference range (maximum 4 points in step 1, and 3 points in step 2). According to the UPSS protocol, each value that exceeded >100% of the upper boundary limit was scored with one point, and those exceeding >150% were scored with two points (maximum score for UPSA: 16 points). EAN/PNS recommended measurements were assessed using EAN/PNS suggested CSA values, and one point was given if the median nerve exceeded >10 mm^2^ at the forearm, >13 mm^2^ upper arm, >9 mm^2^ inter-scalene (trunks) or >12 mm^2^ for nerve roots (Table 1). Additionally, we have provided adjusted upper boundary limits to our laboratory normative values (CSA mean + 2 SD) of the measurements according to EAN/PNS (Table 1). Every measurement that exceeded the upper boundary limits was scored with one point. Despite that this protocol was only being suggested in CIDP patients, we followed the same EAN/PNS suggested protocol for CIDP, AIDP, and MMN patients groups.

The ultrasound-based diagnosis of CIDP was confirmed in patients who scored ≥2 points according to BUS scanning protocol, >5 points according to UPSS protocol, or ≥2 points according to the EAN/PNS recommendations.

The ultrasound-based diagnosis of MMN was established for those who met the condition < 2 on step 1 and ≥1 on step 2 according to the NUP protocol (step 2) or UPSS > 3, UPSC < 1 (no sensory nerves affected) according to the UPSS protocol. The ultrasound-based diagnosis of AIDP was confirmed for patients who had <2 points according to BUS scanning protocol or UPSS < 5, UPSB ≥ 1 UPSC < 1 according to the UPSS protocol.

### 2.3. Statistics

SPSS version 21 (SPSS, Inc., Chicago, IL, USA) and Microsoft Excel for Windows version 2311 (Microsoft, Redmond, WA, USA) were used to analyze data. The Mann–Whitney U test was used for the comparison of age, weight, height between patients and healthy controls. The Kruskal–Wallis test was used to analyze the CSA differences in different disease groups. Post hoc analysis was performed using Bonferroni correction due to multiple t-tests. Data of all cohort CSA size values are presented as mean and the standard deviation (SD) of the median. Sensitivity, specificity, positive, and negative predictive values of protocols were evaluated by cross tables. Spearman’s and Pearson’s tests were used to assess for linear correlations. The strength of the correlation was defined as weak, if r was <0.4, moderate if 0.4–0.6, and strong—if >0.7 [26]. The upper boundary limits for male and female were adjusted according to our laboratory normative values, and were calculated as the CSA mean + 2 SD. Statistical significance was assumed when *p* < 0.05.

## 3. Results

### Demographic Data

We enrolled a total of 189 subjects (105 males and 84 females), comprising 40 CIDP patients, 13 MMN patients, 11 AIDP patients, and 125 healthy controls. The mean ages were 62.49 years (range 37–84 years) for the CIDP patients, 55.92 years (range 32–71 years) for the MMN patients, 68.09 years (range 51–88 years) for the AIDP patients, and 49.02 years (range 25–80 years) for the healthy controls. The characteristics of study subjects are summarized in Table 2. All the patients fulfilled clinical and electrophysiological criteria of typical CIDP according to EAN/PNS [13], and none of them fulfilled criteria of MADSAM.

**Figure 1:**
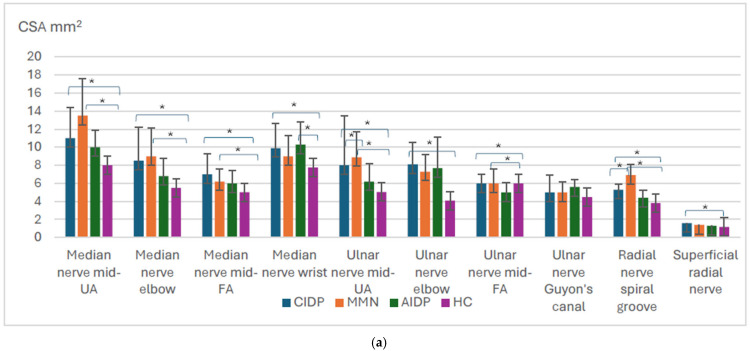

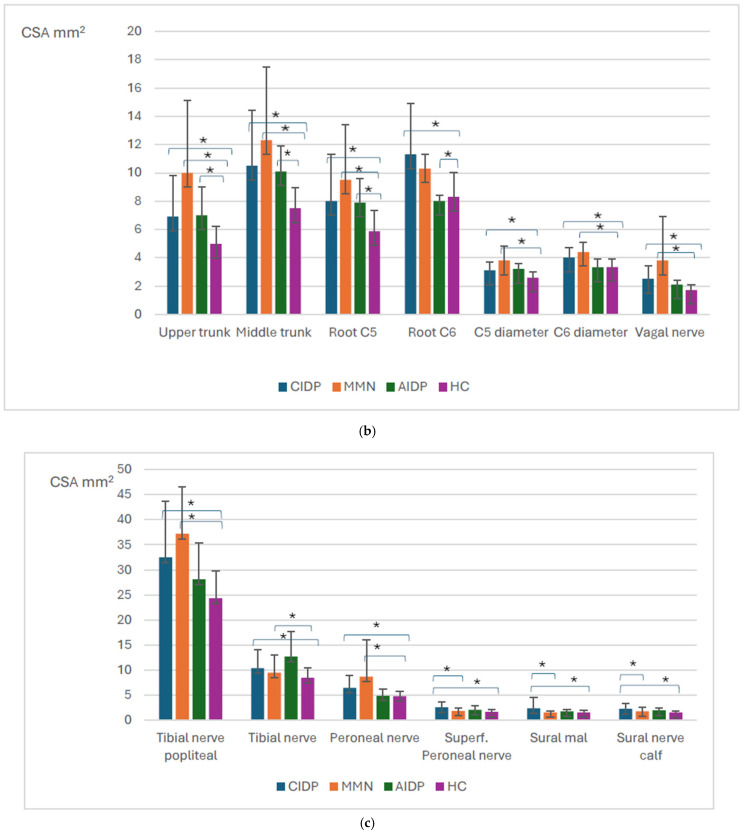
Overview of the significant findings among inflammatory polyneuropathies and healthy controls. Mid—middle, UA—upper arm, FA—forearm. CIDP—chronic inflammatory demyelinating polyneuropathy; MMN—multifocal motor neuropathy, AIDP—acute inflammatory demyelinating polyneuropathy, HCs—healthy controls. Distribution of nerve size according to different polyneuropathy ((**a**) upper limb, (**b**) brachial plexus, and vagal nerve, (**c**) lower limb). Means with standard deviation, *p*-values for the difference between CSA between different polyneuropathies. * *p* value < 0.05.

All measurements of the brachial plexus, vagal nerve, upper limb (except in UN at the Guyons canal), and lower limb nerve measurements were significantly higher in CIDP patients compared to healthy controls (*p* < 0.001) (Figure 1a–c).

**Figure 2:**
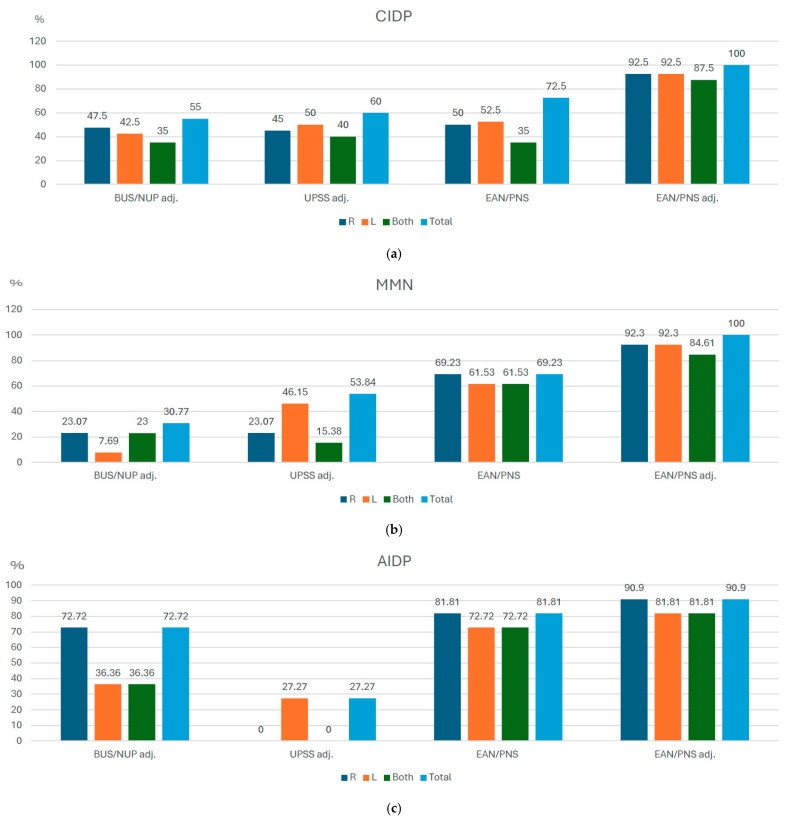
Detection rates in the percentage of inflammatory polyneuropathy cases using unilateral and bilateral measurements in different ultrasound protocols. CIDP—chronic inflammatory demyelinating polyneuropathy; MMN—multifocal motor neuropathy, AIDP—acute inflammatory demyelinating polyneuropathy, BUS adj.—Bochum ultrasound score when cut-off was adjusted according to local reference values. NUP adj.—neuropathy ultrasound protocol when cut-off values were adjusted according to local reference values. EAN/PNS—European Academy of Neurology/Peripheral Nerve Society, *N*—number of patients. (**a**) CIDP, (**b**) MMN, (**c**) AIDP.

A total of 50% of CIDP patients were detected measuring on the right side only, and 52.5% were detected measuring on the left side only, using the EAN/PNS protocol (Figure 2a). The number of positive ultrasound-based diagnoses increased up to 72.9% if patients were measured bilaterally. After the adjustment of the upper boundaries to our laboratory reference values, a total of 92.5% of CIDP patients were detected measuring on the right or left side only according to EAN/PNS protocol. The number of positive ultrasound-based CIDP diagnoses increased to 100% when measuring on both sides. Measurements according to UPSS adj. and BUS adj. protocols detected 60% and 55% of the CIDP cases, respectively. The highest sensitivity (100%) to distinguish CIDP from healthy controls was seen in the EAN/PNS protocol after adjustment (EAN/PNS adj.) to our center’s reference values, and the specificity (90%) was the same as EAN/PNS protocol, BUS/NUP adj., and UPSS adj. protocols. The EAN/PNS adj. protocol had a reduced specificity of 82% (Table 3).

In MMN patients, all measurements of the brachial plexus (except CSA of C6 root), vagal nerve, most of the upper limb (the median nerve at UA, elbow, FA, the ulnar nerve at UA, FA, the radial nerve at SG), and all measurements of the lower limb motor nerves were significantly higher compared to healthy controls, *p* < 0.001 (Figure 1a–c). Measurements of sensory nerves did not differ between the patients and healthy controls (*p* > 0.05). As expected, CIDP patients had higher CSA measurements of all the sensory nerves (except the superficial radial nerve) compared to MMN patients (*p* < 0.005).

There were no differences between CIDP and MMN patients’ nerve measurements, except UN at UA, RN at SG, and the UT of the brachial plexus were higher in MMN patients compared to CIDP (*p* = 0.041, *p* = 0.009, and *p* = 0.006, respectively).

A total of 69.23% of MMN patients were detected when measurements were taken on the right side only, and 61.53% (Figure 2b) when measured on the left side only. The number of detected MMN cases did not increase after data from both sides were pooled together.

EAN/PNS adj. allowed to increase case detection to 100% if measuring bilaterally. The lowest number of cases (30.77%) was achieved by BUS adj. protocol. The highest sensitivity (100%) to distinguish MMN from healthy controls and other inflammatory polyneuropathies was seen in the EAN/PNS protocol using EAN/PNS adj, whilst the UPSS adj. protocol had the highest specificity (96%).

In AIDP patients, all of the measurements of the brachial plexus, except C5 and C6 diameters, were higher compared to healthy controls, *p* < 0.05 (Figure 1a,b).

Also, there was no difference between the unilateral (90.90% measuring one right side) or bilateral (90.90%) measurements (Figure 2c). The lowest number of confirmed AIDP diagnoses was 27.27% of all cases using the UPSS adj. protocol. The highest sensitivity (91%) and specificity (88%) to distinguish AIDP from healthy controls was seen in the EAN/PNS protocol after adjustment.

The CSA of the brachial plexus middle trunk and disease duration was strongly positively correlated in AIDP patients (r_s_ = 0.719, *p* = 0.019). Also, the median nerve at UA and the ulnar nerve at FA negatively weakly correlated with disease duration (r_s_ = −0.341, *p* = 0.033, and r = −0.346, *p* = 0.031, respectively) in CIDP patients.

## 4. Discussion

Our study aimed to compare the diagnostic value (sensitivity and specificity) of well-known ultrasound protocols for different inflammatory polyneuropathies in a single cohort.

This study was performed following original HRUS protocols and EAN/PNS guidelines that suggest using ultrasound as a supportive tool [13,17,18,19,23,27]. Therefore, we adjusted the original protocols according to our laboratory normative values considering the sex difference.

The ultrasound pattern sum score is quite an extensive protocol with three sub-cores (A, B, and C), which encompass upper and lower limb peripheral sensorimotor nerves, cervical roots, vagal nerve, and pure sensory nerves evaluation.

It has been shown that UPS-A had the best sensitivity (80%) and specificity (95.9%) to diagnose CIDP in the analyzed cohort, and UPS-B for AIDP patients fulfilled the criterion with a sensitivity of 63% and a specificity of 90.5% [11]. The readjustment of the UPSS protocol was provided [19], confirming that even more accurate reference values are necessary. Another study [11] showed that the UPS-B sub-score had a sensitivity of 63.2% and a specificity of 90.3% for AIDP diagnosis. We could not confirm these findings, as our study’s results showed quite low UPSS protocol sensitivity (36%) but high specificity (85%) for AIDP ultrasound-based diagnostics. On the other hand, we used more strict conditions, as it was suggested in later publications and patients with AIDP had to fill the conditions UPSB ≥ 1 and UPSC < 1 and UPSS < 5 [15,20].

The BUS algorithm (step 1) with an extension as NUP (step 2 and step 3) has less coverage compared to UPSS. It also encompasses the sensomotor of upper and lower limbs, the sural nerve at the calf solely, and does not include the brachial plexus measurements. Our study’s results were similar to another study where 21 of 24 (87.5%) patients with GBS showed a BUS < 2, while only 33 of 52 (63%) CIDP patients had a BUS ≥ 2 [16,17]. We hypothesized that a long-lasting disease course might change the nerve size and we confirmed this theory showing a negative correlation between disease duration and nerve size, and this corresponds to the previously mentioned study, whose authors [16] theorized that long disease course might be one of the most important factors for different results. Previously, it was shown that the BUS algorithm enabled the diagnosis of 42/49 CIDP patients (85.7%) and 13/15 MMN patients [28], and a score < 2 points was defined for AIDP, showing a sensitivity of 84.2% [17,18]. In our study, UPSS and BUS adjusted protocols showed similar value results, and both detected 55–60% of the CIDP cases. Although we could not reach a high specificity, but the sensitivity of NUP/BUS adjusted protocol was high (90%). Quite recently, the original adjusted Bochum ultrasound score (aBUS) was developed, which can range from 0 to 6, and showed the high specificity of 94% and sensitivity of 59% for distinguishing CIDP from non-inflammatory axonal polyneuropathies. Unfortunately, our research design was developed earlier, so a previously published version of the scale was chosen [29].

In the EAN/PNS guidelines for the diagnosis and treatment of CIDP, the Task Force suggested using ultrasound in adult patients to help diagnose CIDP in patients meeting the diagnostic criteria for possible CIDP. The diagnosis of CIDP could be considered if there is nerve enlargement of at least two sites in proximal median nerves segments and/or the brachial plexus. Despite the fact that this protocol was suggested only in CIDP patients, we have also tried it on MMN and AIDP subgroups. We found that the EAN/PNS protocol was the most valuable not only for CIDP (detected 72.5% of all cases), but this also allowed us to suspected other inflammatory polyneuropathies (69.23% of patients in MMN and 81.81% patients of AIDP). Another important finding was that adjusting the EAN/PNS protocol to our laboratory’s upper boundaries seemed to be the most accurate scale in all the investigated inflammatory polyneuropathy detection.

It is known that, in cases of symmetric neuropathies, unilateral scans might be performed, and in cases of asymmetric neuropathy, bilateral scans are needed [20]. However, even in healthy controls, a side-to-side difference of up to 30% might exist [30]. Our study showed that bilateral measurements might be valuable in patients with inflammatory polyneuropathy even with a symmetrical disease course. The number of positive ultrasound-based CIDP diagnoses increased up to 72.5% (from 52.5% when it was measured unilaterally) when we measured both sides according to the EAN/PNS protocol.

Peripheral nerve ultrasound benefit in AIDP is controversial. Our study was started before the new guidelines for the Guillain–Barré diagnostics and treatment came out [14]. In the new EAN/PNS guidelines, the TF advises against using nerve HRUS as a routine test for the diagnosis of AIDP with typical presentation, and HRUS should be considered only in cases with atypical presentation. It is stated that nerve HRUS might help detect abnormalities in the nerve roots, but this test lacks specificity. In our study, we could not find nerve size differences in most measurement areas, compared to healthy controls. This was different from another study where they found a significant increase in nerve CSA in most of the peripheral nerves measured in AIDP patients compared to a healthy population. The only similarity with this study and ours was that we have found an enlargement of brachial plexus roots (root-dominant nerve enlargement) [31]. Our study results agree that AIDP patients have a “sensory sparing pattern”, but we could not confirm that vagal nerve enlargement would be the hallmark in AIDP, and this is one of the most important reasons why we have found that the UPPS scales suggested an algorithm with such a low sensitivity [15].

In summary, we found that the EAN/PNS protocol, especially after adjusting it to our reference values, was the most valuable tool for determining the condition to be treated, but its specificity was lower. The UPSS and BUS/NUP protocols were more specific and were more accurate in determining CIDP or MMN.

Additionally, we observed some practical considerations that could be useful for clinicians. On average, a full patient examination took around 50 min. Scanning protocols that included the lower limbs were less convenient for patients and more time-consuming due to positioning issues and the difficulty in locating certain nerves. Although the EAN/PNS protocol was generally quick and easy to perform, some difficulties arose in patients with higher body mass index or shorter necks. Scanning according to the BUS protocol, which involved several peripheral nerve entrapment sites, made nerve size differences less evident. Also, we noticed that, in UPSS, converting from CSA values to specific estimates and calculating formulas was quite time-consuming, and some additional training was needed. Evaluating just the CSA value numbers according to reference values seemed much easier. We believe that, in the future, ultrasound might be a helpful tool not only for detecting treatable conditions but also for differentiating between various chronic inflammatory neuropathies that may mimic each other.

## 5. Limitations

This study had some limitations, including the inability to include only treatment-naïve patients, which prevents us from assessing the specific effects of disease duration and treatment on ultrasound findings. Our cohort was relatively small due to the rarity of the conditions being studied and the challenges in recruiting large sample sizes in smaller countries. Additionally, not all AIDP patients were able to undergo intensive ultrasound examination due to physical limitations such as weakness and difficulty changing positions.

## 6. Conclusions

The BUS/NUP and UPSS scales demonstrated lower sensitivity compared to the EAN/PNS suggested protocol across all investigated pathologies, but exhibited high specificity. These findings support the use of BUS/NUP and UPSS protocols for differential diagnosis in various inflammatory polyneuropathies. The EAN/PNS suggested protocol showed the highest sensitivity for CIDP, MMN, and AIDP, making it a valuable tool for detecting treatable conditions. Furthermore, the protocol’s sensitivity and specificity improved when adjusted to our laboratory’s reference values, and bilateral HRUS measurements increased the detection rate in all inflammatory polyneuropathies across the protocols. Considering the results obtained, it is seen that center-specific adjustments can significantly improve the detection of the treatable condition; consequently, we recommend using the EAN/PNS protocol after standard values for individual centers are established.

## Figures and Tables

**Table 1:** Adjusted cut-off estimates and points assigned to each value according to different ultrasound protocols by gender.

	Cut-Off: Mean + 2 SD (mm^2^)		Point
	Male	Female	
**UPS-A. Peripheral sensorimotor nerves (range 0–16)**			
Median nerve upper arm	<11.6	<9.54	0
	≥11.6	≥9.54	1
	≥17.4	≥14.31	2
Median nerve midarm	<7.91	<6.74	0
	≥7.91	≥6.74	1
	≥11.86	≥10.11	2
Median nerve forearm	<6.97	<5.97	0
	≥6.97	≥5.97	1
	≥10.46	≥8.96	2
Ulnar nerve upper arm	<7.42	<6.5	0
	≥7.42	≥6.5	1
	≥11.13	≥9.75	2
Ulnar nerve forearm	<6.06	<5.37	0
	≥6.06	≥5.37	1
	≥9.09	≥8.06	2
Peroneal nerve	<6.54	<6.96	0
	≥6.54	≥6.96	1
	≥9.81	≥10.44	2
Tibial nerve popliteal	<36.28	<33.38	0
	≥36.28	≥33.38	1
	≥54.42	≥50.07	2
Tibial nerve	<12.99	<12.01	0
	≥12.99	≥12.01	1
	≥19.48	≥18.02	2
**UPS-B. Cervical roots and vagal nerve (range 0–3)**			
Root C5	<3.45 mm	<3.33 mm	0
	≥3.45 mm	≥3.33 mm	1
Root C6	<4.56 mm	<4.4 mm	0
	≥4.56 mm	≥4.4 mm	1
Vagus nerve	<2.7	<2.21	0
	≥2.7	≥2.21	1
**UPS-C. Sensory nerves (range 0–3)**			
Sural nerve calf (sural nerve distal calf next to SVV)	<2.22	<2.49	0
	≥2.22	≥2.49	1
Superficial radial nerve	<1.65	<1.62	0
	≥1.65	≥1.62	1
Superficial peroneal nerve	<2.61	<2.42	0
	≥2.61	≥2.42	1
**Bochum ultrasound score (step 1)**			
Ulnar nerve Guyon’s canal	<6.38	<5.91	0
	≥6.38	≥5.91	1
Ulnar nerve upper arm	<7.42	<6.5	0
	≥7.42	≥6.5	1
Radial nerve spiral groove	<5.29	<4.78	0
	≥5.29	≥4.78	1
Sural nerve calf	<2.29	<2.25	0
	≥2.29	≥2.25	1
**NUP step 2**			
Median nerve forearm	<6.97	<5.97	0
	≥6.97	≥5.97	1
Ulnar nerve forearm	<6.06	<5.37	0
	≥6.06	≥5.37	1
Tibial nerve	<12.99	<12.01	0
	≥12.99	≥12.01	1
**NUP step 3**			
Median nerve wrist	<11.85	<10.29	0
	≥11.85	≥10.29	1
Ulnar nerve elbow	<9.55	<8.09	0
	≥9.55	≥8.09	1
**EAN/PNS protocol (EAN/PNS adj.)**			
Median nerve forearm	≤10 (<6.97)	≤10 (<5.97)	0
	>10 (≥6.97)	>10 (≥5.97)	1
Median nerve upper arm	≤13 (<11.6)	≤13 (<9.54)	0
	>13 (≥11.6)	>13 (≥9.54)	1
Upper trunk of brachial plexus	≤9 (≤7.78)	≤9 (≤7.14)	0
	>9 (>7.78)	>9 (>7.14)	1
Middle trunk of brachial plexus	≤9 (≤10.94)	≤9 (≤9.71)	0
	>9 (>10.94)	>9 (>9.71)	1
Root C5	≤12 (≤8.96)	≤12 (≤8.53)	0
	>12 (>8.96)	>12 (>8.53)	1
Root C6	≤12 (≤11.75)	≤12 (≤11.79)	0
	>12 (>11.75)	>12 (>11.79)	1

UPS-A—ultrasound pattern sum score A sub-score; UPS-B—ultrasound pattern sum score B sub-score, UPS-C—ultrasound pattern sum score C sub-score; NUP—neuropathy ultrasound protocol; EAN/PNS protocol—European Academy of Neurology/Peripheral Nerve Society guidelines for diagnosis and treatment of chronic inflammatory demyelinating polyradiculoneuropathy: Report of a joint Task Force-Second revision suggested ultrasound protocol, root C5—fifth cervical root, root C6—sixth cervical root; UPS-A: each value > 100% of the upper boundary limit was scored with 1 point, >150% with 2 points. UPS-B and UPS-C: 1 point was given if the CSA value was higher than reference values (mean + 2 SD) and 0 if the value fit into the normal reference values. BUS/NUP scoring: 1 point was given if the CSA value was higher than the reference values and 0 if the value fit into the normal reference values. EAN/PNS recommended protocol: every measurement that exceeded the upper boundary limits was scored 1 point. Adjusted EAN/PNS upper boundary limits shown in brackets. All the cut-off calculated: mean + 2 SD.

**Table 2:** Demographic and clinical characteristics of the study participants.

Characteristic Variable	Patients Group (*N* = 64)	Control Group (*N* = 125)	*p*
Male, *N*%	65.62	50.4	*p* = 0.018
Age, median, years	61.00	53.00	*p* < 0.001
Age, range, years	32–88	25–80	-
Height, cm	176.14 (9.24) *	173.79 (9.10) *	*p* = 0.091
Weight, kg	81.88 (17.16) *	76.61 (14.56) *	*p* = 0.031
CIDP, *N*%	40	-	-
MMN, *N*%	13	-	-
AIDP, *N*%	11	-	-

* Mean (standard deviation). CIDP—chronic inflammatory demyelinating polyneuropathy; MMN—multifocal motor neuropathy, AIDP—acute inflammatory demyelinating polyneuropathy, *N*—number of cases.

**Table 3:** Sensitivity, specificity, positive predictive value (PPV), and negative predictive value (NPV) of different ultrasound protocols when detecting inflammatory polyneuropathies.

		Sensitivity, %, (95% CI)	Specificity, %, (95% CI)	PPV, %	NPV, %
CIDP	BUS/NUP adj.	55 (47–63)	90 (81–99)	59	89
	UPSS adj.	60 (52–68)	90 (81–100)	72	90
	EAN/PNS	73 (66–80)	90 (81–99)	66	92
	EAN/PNS adj.	100 (98–100)	82 (70–94)	61	100
MMN	BUS/NUP adj.	31 (18–44)	82 (60–100)	11	97
	UPSS adj.	54 (40–68)	96 (86–100)	50	96
	EAN/PNS	69 (56–82)	73 (49–97)	17	97
	EAN/PNS adj.	100 (98–100)	70 (45–95)	18	100
AIDP	BUS/NUP adj.	73 (59–86)	70 (43–97)	13	98
	UPSS adj.	36 (22–51)	85 (64–100)	13	97
	EAN/PNS	82 (70–93)	70 (43–97)	15	98
	EAN/PNS adj.	91 (82–100)	88 (69–100)	32	100

CIDP—chronic inflammatory demyelinating polyneuropathy; MMN—multifocal motor neuropathy, AIDP—acute inflammatory demyelinating polyneuropathy. UPSS adj.—Ultrasound pattern sum score protocol when cut-off were adjusted according to local reference values; BUS adj.—Bochum ultrasound score when cut-off was adjusted according to the local reference values; NUP adj.—Neuropathy ultrasound protocol when cut-off was adjusted according to the local reference values; EAN/PNS—European Academy of Neurology/Peripheral Nerve Society guidelines on the diagnosis and treatment of chronic inflammatory demyelinating polyradiculoneuropathy: Report of a joint Task Force-Second revision suggested ultrasound protocol; EAN/PNS adj.—EAN/PNS protocol adjusted according local reference values, PPV—positive predictive value, NPV—negative predictive value, CI—confidence intervals.

## Data Availability

The original contributions presented in this study are included in the article. Further inquiries can be directed to the corresponding author.

## Abbreviations

HRUS—high-resolution ultrasound, CSA—cross-sectional area, UT—upper trunk, MT—middle trunk, C5— fifth cervical root, C6—sixth cervical root, VN—vagal nerve, MN—median nerve, UN—ulnar nerve, RN—radial nerve, TN—tibial nerve, SN—sural nerve, SFN—superficial fibular nerve, FN—fibular nerve, BUS—Bochum ultrasound score NUP—neuropathy ultrasound protocol, UPSS—ultrasound pattern Sum Score, UA—upper arm, FA—forearm, CIP—chronic inflammatory polyneuropathy, CIDP—chronic inflammatory demyelinating polyneuropathy, MMN—multifocal motor neuropathy, AIDP—acute inflammatory demyelinating polyneuropathy, MADSAM—multifocal acquired demyelinating sensory and motor neuropathy, GBS—Guillain–Barré syndrome, EAN/PNS—European Academy of Neurology/Peripheral Nerve Society, Adjusted (adj.)—when cut-off was adjusted according to local reference values.
